# Association of American Editors

**Published:** 1886-03-20

**Authors:** 


ASSOCIATION OF AMERICAN EDITORS.—This organization (if organization it can be called) is appointed to meet at St. Louis, on Monday evening preceding the meeting of the American Medical Association. It has, we think, been very properly suggested that the meetings be made simply social reunions, rather than devoted to reading of papers and discussions, which, being the work of the medical association, is likely to be reserved to that body, rather than used in this small gathering of editors. The meetings heretofore have attracted little or no interest or attention by the committee of arrangements, and, as a rule, there has been no concert or agreement as to the hall or place of meeting. If the profession or some medical society of the city would take the matter in hand, select a hall, and arrange for the meetings, they might be made occasions of great social pleasure and interest.

